# The Visceral Adiposity Index and Its Usefulness in the Prediction of Cardiometabolic Disorders

**DOI:** 10.3390/nu17142374

**Published:** 2025-07-20

**Authors:** Grzegorz K. Jakubiak, Georgian Badicu, Stanisław Surma, Ewa Waluga-Kozłowska, Artur Chwalba, Natalia Pawlas

**Affiliations:** 1 Department of Pharmacology, Faculty of Medical Sciences in Zabrze, Medical University of Silesia, 41-808 Zabrze, Poland; ewa.walugakozlowska@gmail.com (E.W.-K.); artur.adam.chwalba@gmail.com (A.C.); n-pawlas@wp.pl (N.P.); 2 Department of Physical Education and Special Motricity, Transilvania University of Brasov, 500068 Brașov, Romania; georgian.badicu@unitbv.ro; 3 Department of Internal Medicine and Clinical Pharmacology, Faculty of Medical Sciences in Katowice, Medical University of Silesia, 40-752 Katowice, Poland; stanislaw.surma@sum.edu.pl

**Keywords:** visceral adiposity index, diabetes, dyslipidemia, obesity, metabolic syndrome, metabolically healthy obesity, metabolically unhealthy obesity

## Abstract

Obesity is currently one of the most critical public health problems. Although there is no doubt that obesity is a significant risk factor for developing metabolic disorders, this relationship is not completely straightforward. On the one hand, some patients affected by obesity are metabolically unhealthy, while others are metabolically healthy; on the other hand, metabolic syndrome (MetS) can also occur in people with a normal body weight. A commonly used tool for diagnosing obesity is the body mass index (BMI), but the search for better anthropometric measures is ongoing due to the significant limitations of this measure. Obesity can lead to MetS and cardiovascular diseases (CVDs). Adipose tissue dysfunction is the fundamental mechanism linking obesity and cardiometabolic diseases, which is rooted in the disturbed secretion of adipokines. The visceral adiposity index (VAI) is calculated based on the BMI, waist circumference (WC), blood triglycerides (TG), and high-density lipoprotein cholesterol (HDL-C) concentrations. It was proposed in 2010 as a parameter indicating adipose tissue dysfunction and cardiometabolic risk. According to the research conducted so far, some data confirm a relationship between the VAI value and the risk of developing prediabetes, diabetes, insulin resistance, fatty liver disease, MetS, CVD, and chronic kidney disease. Further research is needed to support the implementation of VAI assessment in routine clinical practice. The purpose of this paper is to present the results of a narrative literature review summarizing current knowledge regarding the VAI and its usefulness in clinical practice for assessing cardiometabolic risk.

## 1. Introduction

### 1.1. Obesity and Its Phenotypes

Obesity is one of the most critical public health problems in the world, and its prevalence continues to increase [1]. To diagnose obesity, it is sufficient to measure body weight and height, which enables the calculation of the body mass index (BMI), defined as the ratio of weight expressed in kilograms to the square of height expressed in meters. For Caucasians, a BMI of at least 30 kg/m^2^ allows us to diagnose obesity [2]. Although obesity is widely regarded as a significant risk factor for developing metabolic syndrome (MetS), this relationship is more complex. Individuals with obesity can be categorized into two types: those who are metabolically healthy (MHO) and those who are metabolically unhealthy (MUO) [3]. Even among individuals with normal body weight, some may develop MetS [4].

It is essential to note that, although simple and commonly used, BMI has several limitations. Notably, it says nothing about the composition of body weight and the distribution of body fat (among other things, it does not reflect the visceral fat content). Slightly more information is provided by the waist circumference (WC) and the waist–hip ratio (WHR), defined as the ratio of the WC to the hip circumference (HC) [5,6]. Therefore, it is necessary to seek parameters derived from anthropometric and, ideally, metabolic parameters that can better indicate the risk of obesity complications. At the same time, such parameters would be much easier to obtain and more widely available in clinical practice than specialized body composition analyses which require special equipment.

In recent years, several new parameters to facilitate the assessment of health risks associated with excess body weight, based on calculated results of anthropometric measurements, have been proposed. Currently, their clinical utility in various contexts and patient populations is being evaluated in scientific studies. The body adiposity index (BAI) [7], the body roundness index (BRI) [8], the body shape index (BSI) [9], and the Belarmino–Waitzberg index (BeWI) [10] are examples of new anthropometric parameters that have been recently proposed. The definitions of these indicators are presented in Table 1. In contrast, the visceral adiposity index (VAI), which is derived based on both anthropometric and biochemical measurements, is a particularly promising tool for the assessment of cardiometabolic risks associated with adipose tissue dysfunction [11].

### 1.2. Metabolic Syndrome

MetS is not a homogeneous disease entity; instead, it is a constellation of risk factors that lead to developing type 2 diabetes mellitus (DM), if not already present, and thus carries a high risk of developing cardiovascular disease (CVD) and cardiovascular events. According to the most commonly used definition, at least three of the following five criteria must be met to diagnose MetS: increased WC, increased serum triglycerides (TG), decreased serum high-density lipoprotein cholesterol (HDL-C), hyperglycemia, and hypertension—or being on pharmacological treatments of these conditions [12]. It should be emphasized, however, that there are different versions of the diagnostic criteria for MetS, which complicates the scientific discourse in this area and makes the population of patients with MetS heterogeneous [4]. The elements mentioned above that comprise the diagnosis criteria are the main components of MetS. In addition, the typical clinical picture of patients diagnosed with MetS often includes the following elements: obstructive sleep apnea [13], hyperuricemia [14], increased sympathetic activation [15], laboratory features of chronic inflammation [16], heart failure [17] (mainly with a preserved ejection fraction [18]), chronic kidney disease [19], fatty liver disease [20], insulin resistance (IR) [21], and polycystic ovarian syndrome (PCOS) [22].

There is growing interest in developing methods to assess subclinical cardiovascular dysfunction, which could enable the identification of patients at increased risk before they manifest clinically evident CVD [23]. Valuable and widely discussed possibilities include assessing arterial stiffness by measuring the pulse wave velocity [24] or evaluating endothelial function using the flow-mediated dilation method [25].

### 1.3. Diabetes Mellitus and Cardiovascular Disease

The number of people living with DM is growing dramatically worldwide. It was 151 million in 2000, 425 million in 2017, and is estimated to reach 629 million by 2045 [26]. DM and its complications generate a substantial economic burden for patients, their families, and healthcare systems [27]. CVDs in the course of atherosclerosis, such as coronary heart disease (CHD), cerebrovascular disease, and peripheral arterial disease (PAD), are the most important cause of morbidity and mortality in patients living with DM [28].

DM accelerates and modifies atherosclerosis development. In PAD, people with DM are more likely to have multi-level occlusion and stenosis, and atherosclerotic lesions occur more commonly in the arteries below the knee [29]. Moreover, DM is a risk factor for developing restenosis after percutaneous transluminal angioplasty with or without stent implantation, which may worsen the prognosis and lead to the necessity of reintervention [30].

### 1.4. Purpose of This Paper

The purpose of this paper is to review the literature and present the most essential information on the current state of knowledge regarding the VAI and its usefulness in clinical practice in assessing cardiometabolic risk.

## 2. Adipose Tissue Dysfunction and Cardiometabolic Disease

Adipose tissue is no longer perceived as a passive energy store. Currently, there is no doubt that it is a metabolically active organ that synthesizes and secretes biologically active substances, known as adipokines, which play a crucial role in maintaining metabolic homeostasis [31,32]. Dysfunction of adipose tissue, the key element of which is disturbed secretion of adipokines, is a widely discussed phenomenon in the context of the pathogenesis of many diseases, such as obesity, MetS [33], CHD [34], PAD [35], chronic kidney disease [36], and fatty liver disease [37]. Of particular interest in scientific research is the importance of perivascular [38] and epicardial [39] adipose tissue in the pathogenesis of CVD.

Currently, over six hundred substances classified as adipokines are known. These include, among others, molecules belonging to hormones, cytokines, and growth factors [40]. Among the adipokines are those that have anti-inflammatory and cardio-protective properties [41]. Table 2 lists selected examples of adipokines. Given the significant role of adipokine dysregulation in the development of IR and cardiovascular dysfunction, efforts are currently focused on developing therapeutic options that modify the activity of specific adipokines [42].

## 3. Visceral Adiposity Index

The concept of the VAI, along with the appropriate mathematical formula to calculate its value, was proposed in 2010 by Amato et al. from the University of Palermo in a paper published in *Diabetes Care*. The VAI is a sex-specific index used that indirectly assesses visceral fat function using easily accessible anthropometric parameters, such as BMI and WC, along with TG and HDL-C measurements. The assumption underlying this discovery was an attempt to develop a parameter that would relate to the distribution and function of adipose tissue and thus correlate with the risk of developing metabolic disorders in the course of obesity, as well as CVD, better than the anthropometric parameters available so far [11].

**Figure 1:**
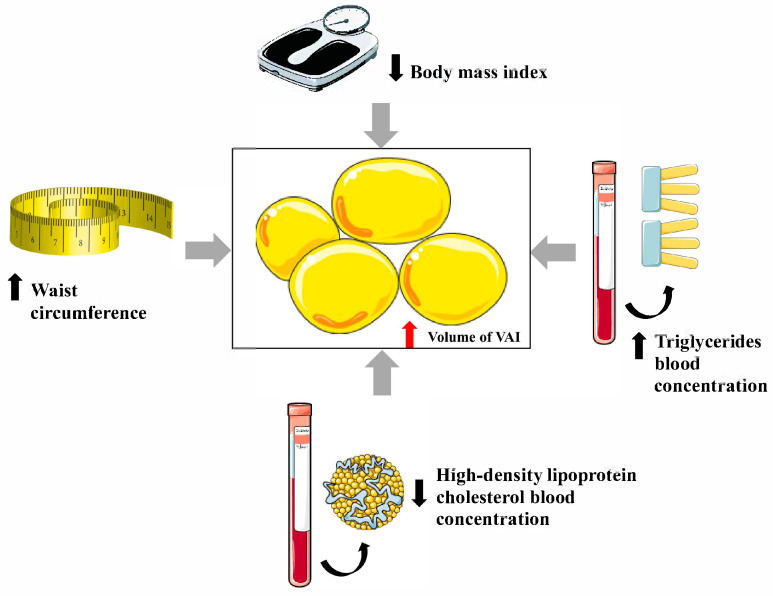
The variables influencing the value of the visceral adiposity index (VAI). This figure was prepared using materials from https://smart.servier.com—CC BY 4.0 license (permission not required).

In Figure 1, the variables influencing the VAI are shown schematically.

The VAI can be calculated based on BMI, WC, TG, and HDL-C using the following formulas:

$$(a)\;\mathrm{Males}:\;VAI=\frac{WC}{39.68+1.88\times BMI}\times \frac{TG}{1.03}\times \frac{1.31}{HDL-C}$$

$$(b)\;\mathrm{Females}:\;VAI=\frac{WC}{36.58+1.89\times BMI}\times \frac{TG}{0.81}\times \frac{1.52}{HDL-C}$$

The VAI is 1.0 for a person with a normal body weight and fat distribution and normal TG and HDL-C values. The equation presented above was designed for Caucasians. However, as explained in the following sections of this publication, many studies have also been conducted using this parameter with representatives of other races.

In 2016, in *Scientific Reports*, Xia et al. introduced the Chinese visceral adiposity index (CVAI) [46] and presented the following equations enabling the calculation of this parameter:

$$\begin{array}{c} (a)\;\mathrm{Males}:\;CVAI=-267.93+0.68\times age+0.03\times BMI+4.00\times WC+22.00\times {log}_{10}TG-16.32\times HDL-C \end{array}$$

$$\begin{array}{c} (b)\;\mathrm{Females}:\;CVAI=-187.32+1.71\times age+4.23\times BMI+1.12\times WC+39.76\times {log}_{10}TG-11.66\times HDL-C \end{array}$$

The mathematical construction of the formula used for calculating the CVAI is therefore quite different from the formula used for calculating the VAI, and it is not merely a difference in the values of the coefficients, as may be expected. However, the CVAI parameter was conceived as a parameter analogous to the VAI, intended for the Chinese population; therefore, it is mentioned here. In this review, we will focus on studies that utilized the VAI.

## 4. Visceral Adiposity Index and Metabolic Disorders

### 4.1. Prediabetes

A total of 70,200 patients without DM and obesity participated in an extensive study performed in China; no association between the VAI and a diagnosis of impaired fasting glucose (IFG) was found. This study did not include an oral glucose tolerance test, giving no information about the relationship between the VAI and the diagnosis of impaired glucose tolerance (IGT) [47]. On the other hand, in another study conducted in China (Gu et al.) on a group of 5457 individuals, it was shown that elevated values of both VAI and WC are independent risk factors for developing prediabetes [48]. The discrepancy between these results might be due to the fact that prediabetes is a subclinical metabolic disorder, which makes the sensitivity of the VAI alone too low for its identification using this method. Including additional variables may improve this sensitivity because, in people with normal WC and increased VAI, the remaining elements that make up the VAI must be abnormal.

In a study performed in India, a median VAI was found to be significantly higher in men with prediabetes when compared to men without abnormalities of carbohydrate metabolism (2.64, IQR: 3.66–1.92 vs. 1.5, IQR: 2.14–1.13; *p* < 0.001), and a similar result was found for women (1.81, IQR: 3.11–1.30 vs. 1.18, IQR: 0.88–1.95; *p* < 0.001). Patients in different categories of body mass participated in this study, and the BMI was significantly higher in patients with prediabetes than in controls of men (28.07 ± 3.59 kg/m^2^ vs. 25.80 ± 3.67 kg/m^2^; *p* < 0.001) and women (28.08 ± 4.18 kg/m^2^ vs. 25.05 ± 3.41 kg/m^2^; *p* < 0.001) [49]. Therefore, it might seem that the BMI has a key influence on the correlation between the VAI and prediabetes, which is not fully confirmed by the results of the study performed in Colombia by Ramirez-Velez et al. People with an average BMI of 26.78 ± 5.02 kg/m^2^ participated in the study. No significant difference in the VAI was found between patients with prediabetes and healthy volunteers when men and women were analyzed together (3.00 ± 3.15 vs. 3.10 ± 3.19; *p* = 0.445), although the mean BMI was significantly higher in subjects with prediabetes (28.13 ± 5.00 kg/m^2^ vs. 26.32 ± 4.94 kg/m^2^; *p* < 0.001). When both genders were analyzed separately, the prevalence of prediabetes was significantly higher in women with VAI above the cutoff, but not in men [50].

Wang et al. recently presented the results of a systematic review and meta-analysis indicating that the VAI could be a predictor of the risk of prediabetes [odds ratio (OR): 1.68; 95% confidence interval (CI): 1.44–1.96]. Importantly, the included studies exhibited publication bias, and significant heterogeneity was observed among the included studies (*I*^2^ = 91.4%; *p* < 0.001) [51].

Based on the cited studies, it can therefore be concluded that although there are some indications of a possible relationship between VAI and the diagnosis of prediabetes, there is no strong evidence for this. Further research is needed to better understand the factors that influence the relationship between the VAI and the risk of prediabetes.

### 4.2. Diabetes

Huang et al. recently published a study based on data analysis from the National Health and Nutrition Examination Survey (NHANES) study (1999–2018). A total of 24,072 individuals were included in the final analysis. It was found that belonging to the next quartiles in terms of the VAI is associated with a significant increase in the risk of carbohydrate metabolism disorders compared to people belonging to the first quartile. OR for quartiles Q2, Q3, and Q4 was, respectively, 1.37 (95% CI: 1.23–1.53), 1.87 (95% CI: 1.65–2.12), and 2.80 (95% CI: 2.33–3.37) [52].

Similar results were obtained by researchers from South Korea analyzing data from 4,058,891 individuals, of whom 15.4% developed DM during a 10-year follow-up period. However, although it has been shown that the VAI indicates the risk of developing DM, the classic anthropometric indicators are superior to it, and this superiority is also dependent on gender. In men, the best indicator was the waist-to-height ratio (WHtR). In women, the choice of the best predictor was also dependent on age. In the youngest women (20–39 years), the best indicator of DM risk was the BMI; in the next age group (40–59 years), it was WHtR; and in the oldest women (60–79 years), the best indicator for assessing the risk of DM was WC [53].

Interesting conclusions were obtained by analyzing the NHANES 2015–2018 study data. A total of 3517 people were included in the final analysis. The VAI was significantly higher in patients with DM than in patients without DM (2.8  ±  2.8 vs. 1.7  ±  1.9; *p*  <  0.0001). Individuals in the fourth quartile of the VAI had the highest risk of developing DM (OR: 2.79; 95% CI: 1.72–4.53; *p*  <  0.0001). Each unit increase in the VAI significantly elevated DM risk (OR: 1.88; 95% CI: 1.69–2.08; *p*  <  0.0001), but only for a VAI below 3.76. When the VAI exceeded 3.76, the association was no longer statistically significant (OR: 1.01; 95% CI: 0.96–1.07; *p*  =  0.6247) [54].

### 4.3. Metabolic Syndrome

According to the results from a study performed by Benbaibeche et al. in Algeria, the VAI is significantly correlated with selected features of MetS in patients affected by obesity, such as WHR (*r* = 0.2; *p* < 0.05), TG (*r* = 0.9; *p* < 0.001), and HDL-C (*r* = –0.6; *p* < 0.001) [55]. According to the results of a study conducted in Peru on a group of 261 people from the general population, the VAI was confirmed to be a good indicator of an increased risk of MetS. The VAI cut-off points at which the area under the curve (AUC) for predicting MetS reached its highest value were 2.57 for women and 1.73 for men, respectively [56]. In another study, conducted on a group of 806 Brazilian rural workers aged 18–59, the VAI was confirmed to be significantly correlated with all components of MetS, such as WC (*r*  =  0.336; *p* < 0.001), systolic blood pressure (*r*  =  0.098; *p* < 0.001), diastolic blood pressure (*r*  =  0.136; *p* < 0.001), fasting plasma glucose (*r*  =  0.124; *p* < 0.001), TG (*r*  =  0.928; *p* < 0.001), and HDL-C (*r* = –0.487; *p* < 0.001) [57].

PCOS is commonly known as a condition associated with an increased risk of developing IR and resulting metabolic disorders [58]. In a study performed by Keyif et al., the VAI was significantly higher in patients with PCOS compared to healthy controls (4.26 ± 3.23 vs. 2.61 ± 1.92; *p* = 0.003). Moreover, among patients with PCOS, the VAI was significantly elevated in individuals with IR and MetS [59].

Jafari et al. conducted a study that assessed the influence of the VAI calculated at the beginning of a 10-year follow-up period on the risk of developing MetS. The analysis included 1084 healthy adults aged 15 to 75, 506 of whom developed MetS during the follow-up period. It was found that an increased VAI significantly increased the risk of MetS [adjusted OR (aOR): 2.57; 95% CI: 1.60–4.12] (OR adjusted for age, sex, job, education, physical activity, smoking, opium consumption, cholesterol, blood pressure, and low-density lipoprotein cholesterol) [60].

### 4.4. Insulin Resistance

Jiang et al. reported the results of the analysis of data from 27,309 individuals (NHANES participants) aged 18 years or older. IR was assessed using the homeostatic model assessment—insulin resistance (HOMA-IR). The study participants were divided into two groups: those with IR features (≥2.73) and those without IR features (<2.73). A positive association was found between the VAI level and IR (OR: 1.28; 95% CI: 1.2–1.37; *p* < 0.001). Among the study participants, 84.1% were diagnosed with DM. However, importantly, the percentage of patients with DM was significantly higher among those without current IR features (93.2%) than among those with current IR features (72.4%) [61]. This suggests that the association between the VAI and IR differs from the association between the VAI and DM. According to another large study conducted in Korea (4922 adults aged 20 or older), HOMA-IR is positively associated with quartiles of the VAI both among people with type 2 DM and among people without type 2 DM analyzed separately [62]. In another study (Štěpánek et al.), 783 individuals with a mean age of 46 who had not previously received treatment with antidiabetic drugs were assessed for features of MetS. A significant positive correlation was found between the VAI and HOMA-IR (*r* = 0.506; *p* < 0.01) [63].

### 4.5. Fatty Liver Disease

The term non-alcoholic fatty liver disease (NAFLD) refers to the presence of the accumulation of more than 5% hepatic steatosis, excluding other liver diseases and in the absence of excessive alcohol consumption [64]. In 2020, a new term was introduced: metabolic dysfunction-associated fatty liver disease (MAFLD) [65], which is reserved for cases of fatty liver disease occurring in patients with coexisting metabolic disorders that otherwise constitute components of MetS.

Based on the analysis of data from 2878 adults from the NHANES study (2017–2020), a one-unit increase in VAI was associated with a significant increase in the risk of NAFLD (OR: 1.40; 95% CI: 1.28–1.52). It should be noted that this study included individuals from the general population belonging to different BMI categories [66]. Another study, which analyzed data from 7522 participants of the NHANES 2003–2018 study, also confirmed a significant positive relationship between VAI and the prevalence of NAFLD, but additionally noted that this relationship was influenced by age, gender, and ethnic differences [67]. A meta-analysis of nine studies by Yi et al. found that VAI is an independent predictor of an increased risk of MAFLD. Specifically, a VAI value > 2.33 indicates an increased risk of MAFLD [68]. On the other hand, in the study by Lajeunesse-Trempe et al., no significant association was found between VAI and the risk of metabolic-associated steatohepatitis (MASH) in morbidly obese patients (OR: 0.99; 95% CI: 0.95–1.04). No significant association was found for the remaining anthropometric parameters with the risk of developing MAFLD or MASH. One of the likely reasons is that in individuals with severe obesity, subcutaneous adipose tissue also significantly influences the anthropometric measurements, which, to a lesser extent than visceral adipose tissue, translates into a risk of developing metabolic disorders [69].

## 5. Visceral Adiposity Index and Cardiovascular Diseases

In a cross-sectional study performed in Poland, the VAI was shown to be correlated (better than the BMI and WC) with a history of myocardial infarction, higher carotid intima-media thickness, DM, prediabetes, and impaired kidney function in women aged 65–74 taken from the general population. Moreover, 2.71 could serve as a cut-off point for high cardiometabolic disease risk. However, no significant correlation was found between the VAI and the ankle–brachial index (ABI) value [70]. According to a study conducted in Egypt, in which 397 patients with type 2 DM participated, the VAI was associated with the 10-year risk of CVD in both men (aOR: 3.18; 95% CI: 1.61–6.26; *p* < 0.001) and women (aOR: 4.16; 95% CI: 1.26–13.68; *p* = 0.019) (the OR was adjusted for sociodemographic and lifestyle factors such as age, education, residence, income, and physical activity; diabetes duration; hypertension; blood lipid profile; and antihyperlipidemic medications) [71].

### 5.1. Cerebrovascular Disease

The problem of the interrelationship between metabolic disorders and developing atherosclerosis does not concern only people affected by obesity. According to a study conducted in China by Zhao et al., being in the third tertile of the VAI is a significant risk factor for asymptomatic intracranial arterial stenosis among people over 40 years of age, but only among people with underweight or normal weight (OR: 3.17; 95% CI: 1.15–8.71), not among individuals who are overweight (OR: 1.12; 95% CI: 0.53–2.35) or with obesity (OR: 2.88; 95% CI: 0.62–13.37). Furthermore, in a separate analysis, the third quartile of the VAI was a significant risk factor for asymptomatic intracranial arterial stenosis among individuals with no abdominal obesity (OR: 2.03; 95% CI: 1.14–3.62), but not among those with abdominal obesity (OR: 2.46; 95% CI: 0.57–10.58) [72]. It should be noted, however, that the mere presence of an insignificant atherosclerotic lesion, although it indicates the condition of the cardiovascular system, does not translate into the occurrence of a cardiovascular event in the future, nor does it authorize the diagnosis of a specific disease entity in the course of atherosclerosis. However, there are also data showing that an increased VAI determines not only an increased risk of stroke, but also a younger age at which this event occurs. Analyzing data from 29,337 NHANES participants, each unit increase in VAI was associated with a significant increase in the prevalence of stroke (OR: 1.12; 95% CI: 1.01–1.24), as well as with an earlier age at stroke occurrence (1.64 years) [*β* = −1.64; 95% CI: (−2.84)–(−0.45)] [73]. Another study, retrospectively conducted in China on a group of 9127 individuals, found that the incidence of stroke increased with each quartile of the VAI (8.6%, 8.7%, 9.2%, and 10.0%; the difference between the fourth and first quartiles was statistically significant) [74].

Interestingly, there are also research results available that indicate the prognostic significance of the VAI in patients suffering from stroke. It turns out that an increased VAI is associated with a significantly higher risk of death within 90 days of illness onset (OR: 5.944; 95% CI: 2.752–12.837). The cutoff value of the VAI for 90-day death was 2.355 (sensitivity, 71%; specificity, 84%). Importantly, no prognostic value was demonstrated for gender, age, and TG values; however, it was confirmed for the National Institutes of Health Stroke Scale (NIHSS) (OR: 1.136; 95% CI: 1.068–1.207) [75].

### 5.2. Coronary Heart Disease

Zhang et al., in a study conducted on a group of 355 patients diagnosed with CHD and 697 controls, noted that the VAI correlates with the risk of developing CHD. Comparing the highest vs. lowest quartile of the VAI, aORs of CHD was 4.44 (95% CI: 2.24–8.82) for women and 4.23 (95% CI: 1.99–9.00) for men (adjusted for age, socioeconomic status, cigarette smoking, alcohol consumption, amount of physical activity, LDL-C, and for women only, menopausal status and hormone therapy use) [76]. According to the results of another team, which involved a group of 33,468 participants in a health screening program, a significant positive correlation was found between the coronary artery calcium score (CACS) and the VAI (*r*  =  0.027; *p*  <  0.001) [77].

Furthermore, data shows that the VAI indicates the risk of developing CHD correlates with the severity of atherosclerotic lesions in the coronary arteries. According to a study conducted by Han et al. on a group of 95 people diagnosed with CHD, the VAI, regardless of other factors, is an indicator of the advancement of atherosclerotic lesions in coronary arteries (OR 18.257; 95% CI: 6.038–30.475) [78]. Similar conclusions were obtained based on data from 253 people with DM, using the SYNTAX scale to assess changes in the coronary arteries [79]. In another study, the VAI was found to correlate better than other anthropometric parameters with the clinical and angiographic severity of CHD in patients with acute coronary syndrome [80].

Interestingly, there is data indicating a relationship between the VAI and the structure of atherosclerotic plaques. Retrospective analysis of data from 782 patients showed that the highest VAI was observed in the group of patients with a mixed structure of plaque (2.8), followed by those with calcified plaque (2.3), and the lowest in those with fatty plaque (1.7) (*p* < 0.001) [81].

However, studies on the relationship between the VAI and coronary artery condition are not entirely unambiguous. Bagyura et al. analyzed data from 460 individuals in a voluntary screening program, and according to their results, the VAI correlates significantly with the coronary artery calcium score (CACS) in men but not in women [82].

### 5.3. Heart Failure

It was found that the risk of heart failure increases significantly with the VAI. Luo et al. analyzed data from the NHANES study (2011–2018) from 8999 people and confirmed the existence of a significant correlation between VAI and the risk of heart failure [83]. Similar conclusions, but enriched with in-depth analyses of various types of heart failure in terms of left ventricular systolic function, were presented by another research group from China. A total of 12,161 participants (aged 54.1 ± 5.8 years) were included in the observation, of whom 15.7% developed heart failure during the 22.5 years of observation. It was shown that a one-unit increase in VAI was associated with an 8% risk of heart failure [hazard ratio (HR): 1.08; 95% CI: 1.06–1.11]. Interestingly, during subgroup analysis, VAI was shown to correlate only with the risk of heart failure with preserved ejection fraction (HFpEF), and not with reduced ejection fraction (HFrEF) [84]. This finding is not surprising because developing left ventricular diastolic dysfunction and subsequent heart failure with preserved left ventricular systolic function is part of the typical clinical picture of the consequences of MetS and its components [85,86,87]. Similarly, according to Zhang et al., a unit increase in the VAI is associated with a 4% increased risk of heart failure (OR: 1.04; 95% CI: 1.02–1.05), without taking into account the type of heart failure in terms of left ventricular systolic function (data from the NHANES 2009–2018 study; 28,764 participants) [88].

The VAI also has some prognostic value in patients diagnosed with heart failure. Wu et al. presented the results of a retrospective observational study analyzing data from 809 people diagnosed with MetS and HFrEF. It turned out that with the increase in the VAI tertile in the studied population, the risk of death from cardiac causes (HR: 3.402; 95% CI: 2.123–5.449) and the risk of readmission due to heart failure symptoms (HR: 4.862; 95% CI: 3.605–6.557) significantly increased during the 24-month follow-up period [89]. Interestingly, researchers from Brazil obtained the opposite results. Based on the observation of 116 people with ischemic heart failure, it was found that patients who had a VAI > 1.21 showed significantly lower risk of death (HR: 0.12; 95% CI: 0.02–0.67) [90]. This seemingly surprising finding fits well with the concept of the so-called obesity paradox, which has already been widely described and discussed in patients with heart failure [91,92,93].

### 5.4. Peripheral Arterial Disease

The results of studies conducted so far indicate a much lesser importance of the VAI in assessing the risk of PAD compared to other CVDs described above. Shi et al. presented the results of a study involving 6615 normal-weight patients with hypertension. PAD was defined by an ABI value of less than 0.9. A significant association was found between the VAI and the risk of PAD in the entire population (OR: 1.55; 95% CI: 1.15–2.10) and among men analyzed separately (OR: 2.12; 95% CI: 1.46–3.07). No significant association was found for women analyzed independently (OR: 1.28; 95% CI: 0.85–1.95). The difference between the prevalence of PAD in individual quartiles of the VAI was not statistically significant (*p* = 0.153) [94]. In another study, no association was found between the VAI and mortality in the population of patients diagnosed with PAD. A 5-year follow-up period included 367 people diagnosed with PAD, of whom 57 (15.5%) died. There was no significant difference between survivors and individuals who died in the follow-up period with regard to the VAI (2.49 ± 1.9 vs. 2.7 ± 2.0; *p* = 0.463) [95].

### 5.5. Atrial Fibrillation

Ozkan et al. presented a retrospective data analysis from 207 patients with obstructive sleep apnea syndrome. They found that the VAI was significantly higher in those diagnosed with atrial fibrillation (AF) compared to those without AF (8, IQR: 5.3–15.5 vs. 6.7, IQR: 3.8–14.6; *p* < 0.001). An elevated VAI significantly increases the risk of AF (OR: 1.81; 95% CI: 1.399–2.341; *p* < 0.001) [96]. Data from 199 patients undergoing coronary artery bypass grafting (CABG) with cardiopulmonary bypass were analyzed in another study. Postoperative AF occurred in 27.6% of patients. An increased VAI was found to be an independent risk factor for AF in this patient population (OR: 1.516; 95% CI: 1.314–2.154; *p* < 0.001) [97].

## 6. Visceral Adiposity Index and Diabetic Kidney Disease

Regarding DM complications, the literature review provides the most data on the association between the VAI and the development of renal dysfunction. In a study performed on 24,871 participants with prediabetes, it was shown that the VAI is significantly positively associated with an increased risk of albuminuria and increased urinary albumin–creatinine ratio [98].

A study in China found that the VAI may indicate an increased risk of developing nephropathy, but not retinopathy, in patients diagnosed with type 2 DM. An increase in the VAI equal to one standard deviation is associated with an increased risk of developing nephropathy (HR: 1.127; 95% CI: 1.050–1.210) [99]. Similar conclusions were obtained in another study conducted in China. Zhao et al. recently presented the results of a retrospective analysis of data from 1817 patients with diagnosed DM. They showed that the VAI is an independent predictor of diabetic kidney disease. Moreover, the prognostic value of the VAI is higher than that of the most commonly used anthropometric indices, such as the BMI, WHR, or WHtR [100].

It was shown (data from the Action to Control Cardiovascular Risk in Diabetes (ACCORD) trial participants) that in patients with DM, a VAI ≥ 2.6 compared to VAI < 2.6 is associated with a significantly increased risk (aHR: 1.09; 95% CI: 1.01–1.18) of composite nephropathy outcome, defined as (1) serum creatinine doubling or a > 20 mL/min decrease in the estimated glomerular filtration rate (eGFR), (2) development of macro-albuminuria, or (3) renal failure or end-stage kidney disease (dialysis) or serum creatinine > 3.3 mg/dl; (HR adjusted for age, sex, race, lipid therapy, smoking and drinking status, history of CVDs, years of DM, eGFR at baseline, cumulative average systolic blood pressure, glycated hemoglobin, low-density lipoprotein cholesterol, cumulative average WC, BMI, HDL-C, and TG) [101].

## 7. Conclusions

The literature review results indicate that the VAI is of significant interest in scientific research. Notably, several studies have already been conducted on large patient populations evaluating the importance of the VAI in assessing the risk of various cardiometabolic diseases. Although the studies are heterogeneous in terms of methodology and study populations, some data indicate that the VAI could be a good and useful marker of increased cardiometabolic risk.

In summary, the results suggest that the VAI may become a valuable parameter in the future for assessing the risk of selected cardiometabolic diseases. However, further studies are needed to support its integration into specific algorithms and recommendations, making it easier to use in everyday clinical practice.

The most important findings of this literature review are summarized in Table 3.

## Figures and Tables

**Table 1:** The definitions of selected parameters based on anthropometric variables [7,8,9,10].

Parameter	Definition
BAI	$BAI=\frac{HC}{H^{1.5}}-18$
BRI	$BRI=364.2-365.5\sqrt{1-{\left(\frac{WC}{\pi \times H}\right)}^{2}}$
BSI	$BSI=\frac{WC}{\sqrt[{3}]{(BMI{)}^{2}}\times \sqrt[{2}]{H}}$
BeWI	$male\;BeWI=-48.8+0.087\times AC+1.147\times HC-0.003\times {HC}^{2}-7.195$ $female\;BeWI=-48.8+0.087\times AC+1.147\times HC-0.003\times {HC}^{2}$

Abbreviations: BAI—body adiposity index; BRI—body roundness index; BSI—body shape index; BeWI—Belarmino–Waitzberg index; HC—hip circumference; H—height; WC—waist circumference; BMI—body mass index; AC—abdominal circumference.

**Table 2:** Selected examples of both pro-inflammatory and anti-inflammatory cytokines [41,43,44,45].

Pro-Inflammatory Adipokines	Anti-Inflammatory Adipokines
leptin, visfatin, chemerin, resistin, osteopontin, retinol-binding protein 4 (RBP-4), angiopoetin-like protein (ANGPTL), tumor necrosis factor-α (TNF-α), interleukin-6 (IL-6)	adiponectin, omentin, apelin, nesfatin-1, isthmin-1, secreted frizzled-related proteins (SFRPs), C1q/TNF-related protein (CTRP) family, myeloid-derived growth factor (MYDGF)

**Table 3:** The most important findings of the literature review.

Subject Matter	The Most Important Conclusions
Relationship between the visceral adiposity index (VAI) and the risk of metabolic disorders	The studies conducted so far do not provide convincing evidence of a relationship between the VAI and the risk of prediabetes [47,48,49,50,51].
	There is a clear positive association between the VAI and the risk of diabetes mellitus [52,53,54].
	The VAI is valuable in assessing the risk of metabolic syndrome [55,56,57,59].
Relationship between the VAI and cardiovascular diseases	A higher VAI indicates an increased risk of asymptomatic intracranial arterial stenosis [72], risk of stroke [73,74], and a worse prognosis [75].
	According to most studies, the VAI correlates well with the incidence of coronary heart disease (CHD) and the advancement of atherosclerotic lesions [76,77,78,79,80,81,82].
	An elevated VAI indicates an increased risk of heart failure, especially with preserved ejection fraction [83,84,88]. The prognostic value is ambiguous [89,90].
	The VAI has no evidence of utility in assessing the risk of peripheral arterial disease (PAD), nor is there a significant prognostic value of the VAI in patients already diagnosed with PAD [94,95].
	The VAI correlates with risk of atrial fibrillation in patients with obstructive sleep apnea [96] and in patients undergoing surgical myocardial revascularization [97].
Relationship between the VAI and diabetic kidney disease	The VAI is closely related to the risk of diabetic kidney disease [98,99,100,101].
